# Dysentery

**Published:** 1859-09

**Authors:** 


### DYSENTERY.

Multitudes of lives are lost by ignorance of the nature of simple diseases at their first appearance. Few know the essential difference between diarrhœa, which is ordinarily a trivial disease, and dysentery, which is often a speedily fatal malady.

Diarrhœal discharges always afford a feeling of relief, without pain necessarily, or blood. Dysentery, on the contrary, is always attended with painful gripings, with distressing and ineffectual straining, and more or less blood.

In dysentery, too much blood is thrown in upon the bowels, and nature attempts to relieve herself by passing it off. If she is interfered with, and the mouths of the little tubes which are throwing off the blood are suddenly closed up by styptics, such as alum, or sugar of lead, or logwood and the like, or by opiates in any form, which, in effect, operate in the same way, then the blood takes another direction and goes to the brain, oppresses it, weighing down all the powers of life, and there is delirium, stupor, death. These are vital facts, known to all educated physicians, and yet the very first effort made in the cure of dysentery is to stop the blood, and its diminution is considered encouraging by the ignorant. There is intolerable heat and thirst in dysentery; this heat extends from the tip of the tongue all through the body; this attracts more blood, just as a mustard plaster attracts blood. The true cure is to cool the internal surface of the bowels, and nature calls ravenously for this cooling; yet every swallow of ice water increases the pain, but ice broken up in pieces small enough to be swallowed whole, and taken to the fullest desire and capacity of the patient, cools off the inner surface of the intestinal canal, just as certainly as small lumps of ice constantly placed on a red hot iron surface, will at length cool it. As an aliment, raw beef in the shape of mince-meat, given in quantities of two table-spoon fulls four times a day, at equal intervals, facilitates the cure, while it sustains the patient. These things are advised as domestic expedients, only until a physician can be had.

Dysentery is very generally caused by a sudden cooling of the skin, especially after exercise; or in weakly persons a sudden change in the weather is all sufficient, particularly when with a greater coolness there is a raw dampness in the atmosphere. Thus it is that this serious ailment is so common in the fall of the year; mid-day being hot, and the cool nights closing abruptly the pores of the skin, which the heats of the day had relaxed. The best preventives are wearing woolen flannel shirts, and having fires kindled in the family room at sundown, especially in valley situations, and those otherwise damp, beginning these on the first cool night of the fall.

